# Saquinavir Ameliorates Liver Warm Ischemia-Reperfusion-Induced Lung Injury via HMGB-1- and P38/JNK-Mediated TLR-4-Dependent Signaling Pathways

**DOI:** 10.1155/2017/7083528

**Published:** 2017-12-26

**Authors:** Zhuang Yu, Yao Tong, Renlingzi Zhang, Xibing Ding, Quan Li

**Affiliations:** Department of Anesthesiology, Shanghai East Hospital, School of Medicine, Tongji University, Shanghai 200120, China

## Abstract

Liver ischemia and reperfusion (I/R) induce local and distant tissue injuries, contributing to morbidity and mortality in a wider range of pathologies. This is especially seen under uncontrolled aseptic inflammatory conditions, leading to injury of remote organs, such as lung injury, and even failure. Saquinavir (SQV) is a kind of HIV protease inhibitor that possesses an anti-inflammatory property. In this study, we investigated whether SQV suppresses Toll-like receptor 4- (TLR4-) dependent signaling pathways of high-mobility group box 1 (HMGB1) and P38/JNK, conferring protection against murine liver I/R-induced lung injury. To investigate our hypothesis, C57BL/6 mice and TLR4 knockout mice (TLR4^−/−^) were used to perform the study. SQV administration markedly attenuated remote lung tissue injury after 1-hour ischemia and 6-hour reperfusion of the liver. To our expectation, SQV attenuated I/R-induced lung edema, hyperpermeability, and pathological injury. The beneficial effects of SQV were associated with decreased levels of circulating and lung tissue inflammatory cytokines, such as IL-6, IL-1*β*, TNF-*α*, and iNOS. The protective effect of SQV was also associated with decreased lung tissue expression of HMGB1, TLR-4, and p-P38/JNK, but not p-ERK in wild-type liver I/R mice. Overall, this study demonstrated a new role of SQV, facilitating negative regulation of HMGB1- and P38/JNK-mediated TLR-4-dependent signaling pathways, conferring protection against liver I/R-induced lung injury.

## 1. Introduction

Liver I/R causes systematic sterile inflammation that not only damages the local organ itself but also led to uncontrolled systemic inflammation, resulting in remote organ injury and morbidity [1–3]. Several studies have reported that the lungs are easily susceptible to damage during liver I/R [4, 5].

Toll-like receptors (TLRs) are the most thoroughly studied sentinel pattern recognition receptors (PRRs) [6–8]. Recently, we [9] and others [10–12] have identified that TLR4 plays an indispensable role in the liver I/R injury. High-mobility group box1 (HMGB1) is originally discovered as a nuclear protein that is released to the cytosol and even extracellular space in response to specific conditions [13, 14]. Of note, HMGB1 has been identified as an endogenous TLR4 ligand [15, 16] and evidences suggest that HMGB1-TLR4 activation can lead to immunopathological disorders in the acute state, such as I/R [10], hemorrhagic shock [17], and trauma [18]. In addition, TLR4 also participates not only in the recognition of HMGB1 but also in its release [15]. Mitogen-activated protein kinases (MAPKs) are signaling components that significantly convert extracellular stimuli into cellular responses [19, 20]. Furthermore, studies have demonstrated that HMGB1 regulates inflammatory responses through MAPK signaling pathways [14, 19].

SQV is the first-generation HIV protease inhibitor that possesses anti-inflammatory characteristics. Gero et al. and Pribis et al. [21, 22] recently identified SQV in a medium-throughput screening assay. Results revealed that SQV enhanced the survival of mice after cecal ligation and puncture (CLP) and reduced warm I/R injury in the liver. Furthermore, the study proved that SQV inhibited HMGB1-driven inflammation via targeting the interaction of TLR4/MyD88 [22].

Thus, we explored whether SQV could protect lung inflammation and injury induced by liver I/R via the TLR4/HMGB1 signaling pathway.

## 2. Materials and Methods

### 2.1. Animals

Male wild-type mice (C57BL/6; 8–12 weeks old) were bought from Shanghai Laboratory Animal Co. Ltd. (SLAC, Shanghai, China). TLR4 knockout (TLR4^−/−^) mice were kindly provided by Dr. Timothy R. Billiar (University of Pittsburgh, USA). All mice were fed under a 12 h day/night cycle under specific pathogen-free atmosphere at the Shanghai Tongji University. Animal protocols were approved by the Ethics Committee of the University of Tongji, and the experiments were performed in accordance with the National Institutes of Health Guidelines for the Use of Laboratory Animals.

### 2.2. Preparation of the Hepatic I/R Injury Model

The model of partial hepatic warm I/R was prepared as described previously [9]. Briefly, after a suitable level of anesthesia (100 mg/kg ketamine and 10 mg/kg xylazine) has been attained, a midline laparotomy was performed and an atraumatic clip was used to interrupt the arterial and the portal venous blood supply to the left lateral and median lobes of the liver. Sham control mice underwent the same procedure without vascular occlusion. Mice suffered for 1 hour with partial hepatic ischemia. Then, the mice were sacrificed after 6 hours or 12 hours of reperfusion, and lung tissues and blood samples were used for analysis. SQV (0.5 mg/kg) or nontoxic DMSO was pretreated 1 h before ischemia and again at the time of reperfusion. As well, JNK inhibitor (10 mg/kg SP600125; Calbiochem) and P38 inhibitor (10 mg/kg SB203580; Calbiochem) were administrated as SQV.

### 2.3. Histological Examination

The right superior lobe of the lung of each model was used for histological hematoxylin eosin (H&E) staining. To evaluate the degree of lung injury, the histological alterations of lung parenchyma were quantitatively graded on a scale from 0 to 2 (by alveolar and capillary edema, intravascular and peribronchial influx of inflammatory cells, thickness of the alveolar wall, and hemorrhage) [23].

### 2.4. The Alveolar-Capillary Permeability by Evans Blue Albumin (EBA)

Alveolar-capillary permeability was estimated with EBA according to our previous description [23, 24]. EBA was administered through the vena jugularis externa 1 h before sacrificing all models, and then, the lung tissue was reserved to do further research.

### 2.5. The Wet/Dry Ratio (W/D)

Lung edema was measured by tissue W/D ratio. After dissection, right lung samples were weighed and then placed in a drying oven at 67°C until a constant weight was obtained.

### 2.6. Measurement of Cytokines

Serum and BALF levels of TNF-*α*, IL-6, IL-10, and macrophage inflammatory protein- (MIP-) 2 were determined by using enzyme-linked immunosorbent assay (ELISA, R&D Systems, USA).

### 2.7. RNA Extraction, Reverse Transcription PCR, and Quantitative Real-Time PCR Total

RNA was extracted from lung tissues using TRIzol reagent (Sigma-Aldrich) according to the standard protocols [23]. All the primers were synthesized by Sangon Biotech (Shanghai, China). Primers sequence was presented in Table 1.

### 2.8. Western Blot Analysis

Western blotting for HMGB1, TLR4, p-P38/P38, and p-JNK/JNK in lung tissues was performed as a standard protocol [9]. Membranes were blocked with 5% skimmed milk, incubated with mouse primary antibody against HMGB1 (Abcam, USA), TLR4 (Abcam, USA), p-JNK/JNK (CST, USA) and P38 (AR, USA) and p-P38 (SAB, USA), and I*κ*B*α* and p-I*κ*B*α* (CST, USA) overnight, and then incubated with secondary antibody (Licor Biosciences, USA).

### 2.9. TUNEL Assay

This was performed to examine the apoptotic cells in the lung using the In Situ Cell Death POD kit (Roche, USA) according to the standard protocol [25].

### 2.10. Immunohistochemistry (IHC)

IHC was performed for both macrophage and neutrophil infiltrations using CD11b and Ly6G antibodies (Servicebio, China) [26].

### 2.11. Statistical Analysis

Results were presented as means ± standard error of the mean (SEM) of at least three repeating experiments. Statistical analysis was performed using GraphPad Prism 5.0 (GraphPad Software Inc., San Diego, CA). Analysis was performed using Student's *t*-test or one-way ANOVA. *P* < 0.05 was considered to be statistically significant.

## 3. Results

### 3.1. Liver I/R Mediates Local Hepatic and Remote Lung Injury

**Figure 1:**
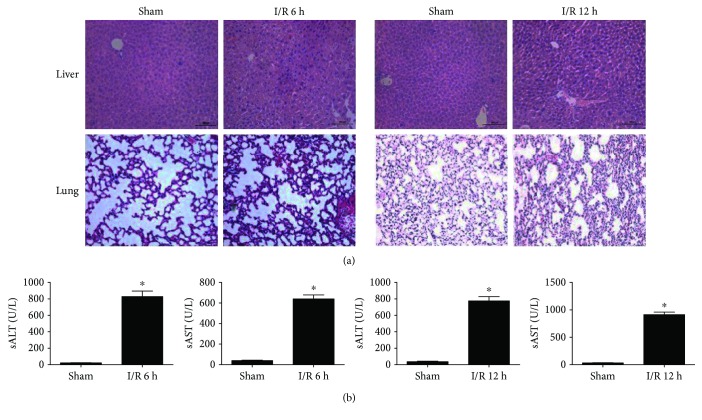
Liver I/R leads to local hepatic and remote lung injury. Liver and lung sections stained with H&E after liver reperfusion at 6 h or 12 h (a). Concentrations of AST and ALT were detected following liver reperfusion at 6 h or 12 h (b). GraphPad values are presented as mean ± SEM. ^∗^ *P* < 0.05 versus the Sham group.

To determine whether reperfusion time could affect the damage degree, we performed H&E staining of lung and liver tissues in both I/R and Sham groups with/without 1 h of liver ischemia following 6 h or 12 h reperfusion. Results demonstrated that the inflammatory cells and structural damage are easily observed both in the liver as well as lung tissues (Figure 1(a)). In addition, the degree of liver cell injury was measured by serum aspartate aminotransferase (AST) and alanine aminotransferase (ALT) levels. Results showed that the levels were significantly increased in the I/R group when compared with Sham group (Figure 1(b)). However, there were no significant differences between reperfusion at 6 h and 12 h. Hence, 6 h was used as the reperfusion time for the following experiments.

### 3.2. SQV Can Improve the Lung Injury Induced by Liver Warm I/R

**Figure 2:**
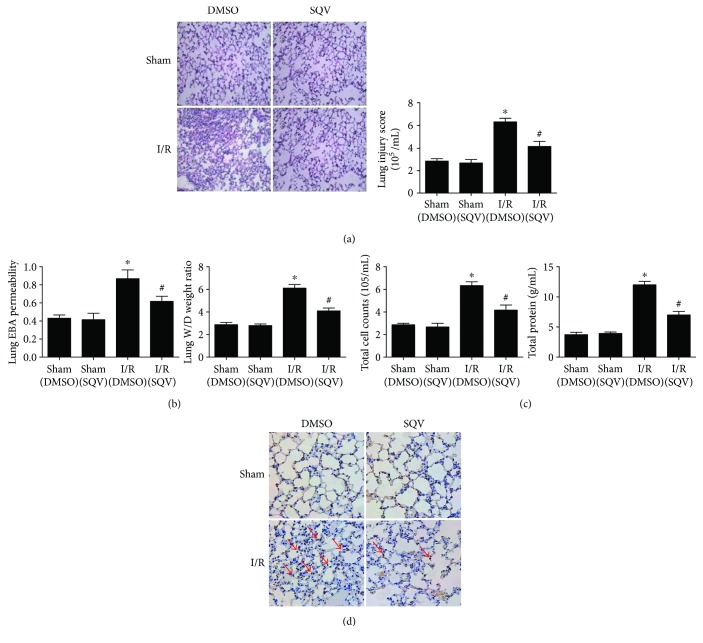
SQV could improve the lung injury induced by liver warm I/R. H&E staining and histological alterations of lung parenchyma were graded (a). Degrees of lung permeability and edema were reflected by EBA and W/D ratio (b). The total cell counts and protein concentration in BALF (c). Apoptotic assay (Tunel^+^) was performed in the lung tissues of mice with or without SQV treatment after liver I/R (d). GraphPad values are presented as mean ± SEM. ^∗^ *P* < 0.05 versus the Sham group; ^#^ *P* < 0.05 compared to the liver warm I/R group.

H&E staining was used to evaluate the general morphology of lung tissues. Compared with the Sham group, lung injury score displayed a higher level in the I/R group, but a markedly decreased level in the I/R + SQV group versus the I/R group (Figure 2(a)). On the other hand, the EBA and W/D ratio showed significant improvement of lung permeability and edema by SQV (Figure 2(b)). Furthermore, total cell counts and protein levels in the I/R group were significantly higher than those in the Sham group, and all these factors in the I/R + SQV group were significantly decreased compared with those in the I/R group (Figure 2(c)). Finally, apoptosis (Tunel^+^) assay was performed, which showed reduced apoptotic rate in lung tissues after SQV treatment in I/R mice (the red arrows, Figure 2(d)). Collectively, these results indicated that SQV has a positive effect on attenuating lung injury induced by liver warm I/R.

### 3.3. SQV Regulates Macrophages in the Lungs after Liver I/R

**Figure 3:**
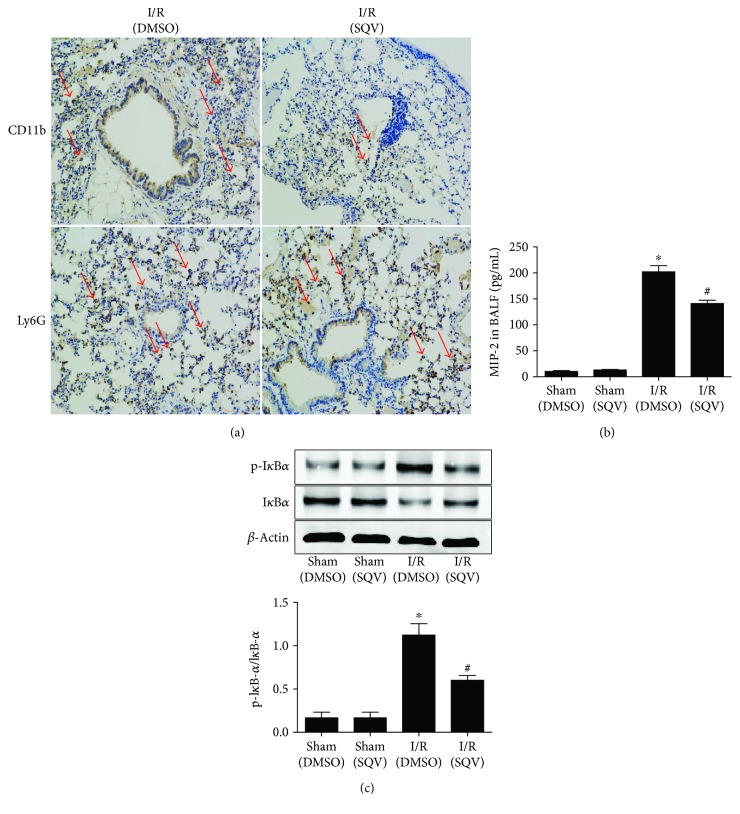
SQV regulates macrophages in the lung after liver I/R. IHC was used to stain either macrophages (CD11b^+^) or neutrophils (Ly6G^+^) in the lung tissue after SQV treatment (a). As well, the MIP-2 levels in BALF were tested by ELISA (b). I*κ*B*α* and p-I*κ*B*α* activation of local lung tissue from all groups was assessed by Western blot (c). GraphPad values are presented as mean ± SEM. ^∗^ *P* < 0.05 versus the Sham group; ^#^ *P* < 0.05 compared to the liver warm I/R group.

Both macrophages (CD11b^+^) and neutrophils (Ly6G^+^) play crucial roles in the secretion of various cytokines. IHC indicated that macrophages were mainly interfered by SQV treatment in the liver I/R mice (the red arrows marked, Figure 3(a)). In addition, MIP-2 levels in the bronchoalveolar lavage fluid (BALF) were downregulated in I/R mice with SQV treatment (Figure 3(b)). Moreover, the NF-kB pathway plays a major role in promoting the release of cytokines; changes to endogenous NF-kB can be measured by the I*κ*B*α* degradation, which begins degradation when NF-kB is activated [27]. Western blot analysis (Figure 3(c)) exhibited a significant upregulation of I*κ*B*α* in the lungs of I/R mice when treated with SQV.

### 3.4. SQV Suppresses Proinflammatory Cytokines and Upregulates the Release of Anti-Inflammatory Cytokines in the Serum and BALF following Liver Warm I/R

**Figure 4:**
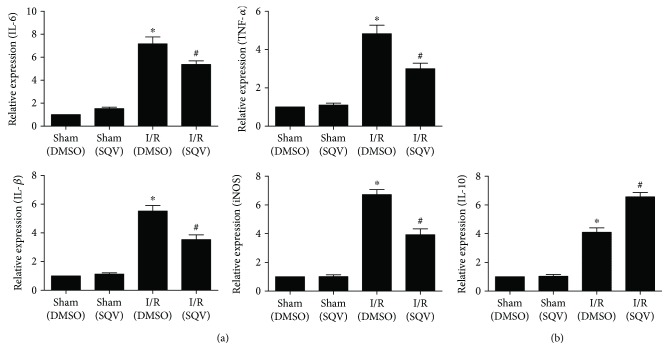
SQV suppresses the level of proinflammatory cytokines and upregulates the level of anti-inflammatory cytokines in the serum and BALF following liver warm I/R. ELISA was performed to check the concentration of cytokines, such as IL-6, TNF-*α*, and IL-10 in BALF (a) and serum (b). GraphPad values are presented as mean ± SEM. ^∗^ *P* < 0.05 versus the Sham group; ^#^ *P* < 0.05 compared to the liver warm I/R group.

To test whether SQV affects the production of cytokines, ELISA was performed. BALF proinflammatory cytokines, TNF-*α*, and IL-6 were increased in the I/R group compared with the Sham group, but their levels were markedly decreased in the I/R + SQV group. In addition, IL-10, as an anti-inflammatory factor, was increased in BALF in the I/R group compared to the Sham group. Otherwise, the IL-10 levels were much higher in the I/R + SQV group compared to the I/R group (Figure 4(a)). Similar results were observed in the serum (Figure 4(b)).

### 3.5. SQV Affects Cytokines at the Gene Transcriptional Level in Lung Tissues after Liver I/R

**Figure 5:**
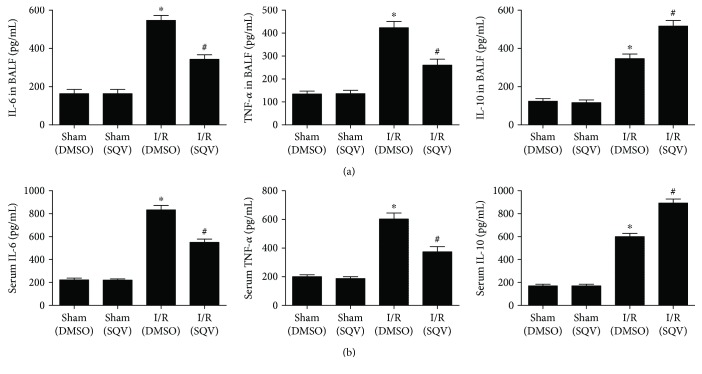
SQV inhibits transcriptional expression of proinflammatory factors and enhances transcriptional expression of anti-inflammatory factors in lung tissue after liver I/R. qPCR was performed to check the expression of proinflammatory factors of IL-6, TNF-*α*, IL-1*β*, and iNOS (a) and anti-inflammatory factor, IL-10 (b). GraphPad values were presented as means ± SEM. ^∗^ *P* < 0.05 versus the Sham group; ^#^ *P* < 0.05 compared to the liver warm I/R group.

qPCR was performed to further investigate the effects of SQV on the production of various factors. To meet our expectation, proinflammatory factors, such as TNF-*α*, IL-6, IL-1*β*, and iNOS, were all significantly increased in the I/R group compared to the Sham group, but a significant decrease in the transcription of these genes was observed compared to the I/R + SQV group (Figure 5(a)). Of course, the expression of IL-10 followed the results of its protein level in serum and BALF (Figure 5(b)).

### 3.6. SQV Attenuates HMGB1 and TLR4 Expression Levels and Affects Phosphorylation of MAPK Signaling Proteins in the Lung Tissues after Liver I/R

**Figure 6:**
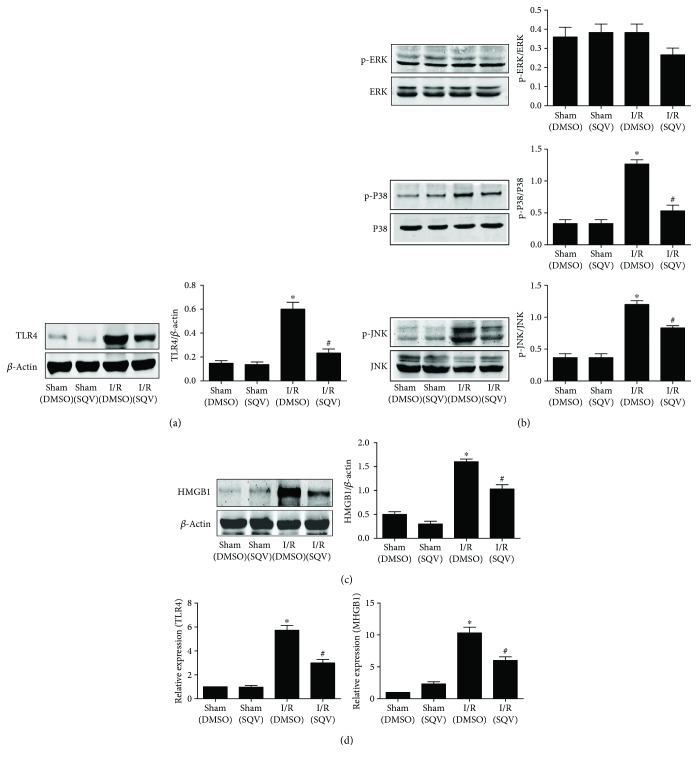
SQV attenuates HMGB1 and TLR4 expressions and affects phosphorylation of MAPK signaling proteins in the lung tissue after liver I/R. Western blot was used to reflect the protein levels of TLR4 (a) and HMGB1 (b); the phosphorylation of ERK, P38, and JNK were also checked by immunoblotting analysis (d). qPCR was performed to confirm the expression level of TLR4 and HMGB1 (c). GraphPad values are presented as means ± SEM. ^∗^ *P* < 0.05 versus the Sham group; ^#^ *P* < 0.05 compared to the liver warm I/R group.

Western blot and qPCR were performed to investigate the underlying mechanism of SQV. Results revealed reduced inflammation of liver I/R after SQV treatment. TLR4 (Figure 6(a)) and HMGB1 (Figure 6(b)) protein levels were distinctively increased in the I/R group compared to the Sham group and visually decreased in the I/R + SQV group by immunoblotting. Similar results were confirmed by qPCR (Figure 6(c)). Phosphorylation of MAPK signaling proteins was also measured (Figure 6(d)). Interestingly, phosphorylated JNK and P38 were markedly decreased in the I/R + SQV group compared to the I/R group, but showed no change in the ERK phosphorylation. Overall, these results implied that SQV could mediate the TLR4/HMGB1-JNK and P38 MAPK signaling pathways to improve lung injury after liver I/R.

### 3.7. HMGB1 and P-JNK/P38 Markedly Attenuate in Lung Tissue from TLR4-Targeted Mice

**Figure 7:**
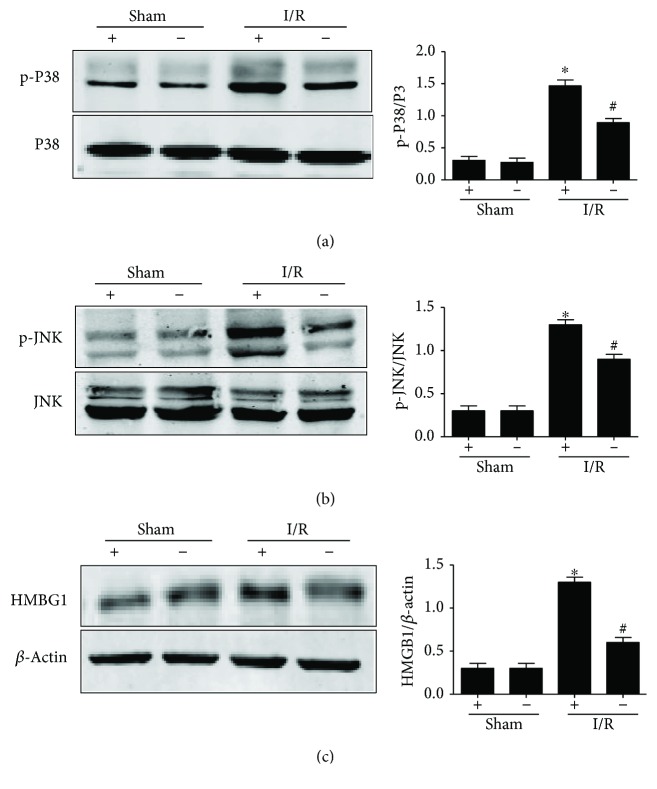
HMGB1 and p-P38/JNK were markedly attenuated in the lung tissues in TLR4-targeted mice. Lung homogenates of all groups extracted the proteins to check the phosphorylation of P38 (a) and JNK (b); HMGB1 level was also checked (c). GraphPad values are presented as mean ± SEM. ^∗^ *P* < 0.05 versus the Sham group; ^#^ *P* < 0.05 compared to the liver warm I/R group.

Previous studies suggested that the TLR4 system may play a key role in HMGB1-mediated hepatic I/R injury, and TLR4-targeted mice were not affected by administration of rHMGB1 or neutralizing antibodies to HMGB1 in liver I/R compared with TLR4-intact mice [10]. So, we used TLR4^−/−^ mice to explore whether TLR4 would change the release of HMGB1 and decrease the phosphorylation of JNK/P38 MAPKs in lung tissues after liver I/R. Western blot analysis was performed on lung lysates from TLR4^−/−^ mice subjected to liver I/R or not (Figure 7). Following 1 h of warm ischemia and 6 h of reperfusion, HMGB1 protein expression demonstrated no upregulation in the TLR4^−/−^ mice compared to wild-type mice (Figure 7(c)). Also, P38 (Figure 7(a)) and JNK phosphorylation (Figure 7(b)) in TLR4^−/−^ mice with liver I/R were reduced than TLR4-intact mice.

### 3.8. P38 and JNK Inhibitors Can Attenuate the Lung Injury Induced by Liver I/R

**Figure 8:**
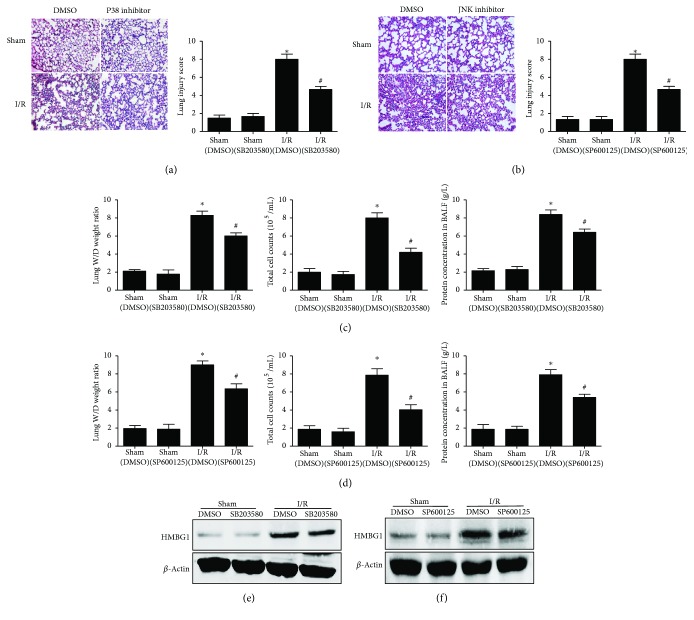
P38 and JNK inhibitors could make little difference on HMGB1 protein level in lung tissue but still relieve the lung injury induced by liver I/R. H&E staining and histological alterations of lung parenchyma were shown (a, b). Degree of lung edema and permeability were reflected by W/D ratio, total cell counts, and protein concentration in BALF (c, d). HMGB1 from both inhibitors with or without treatment of lung tissue was also checked (e, f). GraphPad values are presented as mean ± SEM. ^∗^ *P* < 0.05 versus the Sham group; ^#^ *P* < 0.05 compared to the liver warm I/R group.

Finally, we investigated whether P38/JNK MAPKs were downstream molecules of TLR4/HMGB1-denpendent signaling pathways. Wild-type mice with liver I/R pretreated with SB203580 or SP600125 were taken as the experimental group. Lung H&E staining was performed (Figures 8(a) and 8(b)). Results showed that both inhibitors were markedly improved in the I/R + SQV group compared to the I/R group. Furthermore, we detected the lung W/D ratio, total cell counts, and protein concentrations in BALF to conform our expectations. Results demonstrated that both inhibitors could relieve lung injury induced by liver I/R (Figures 8(c) and 8(d)). Importantly, the HMGB1 protein level was not markedly decreased by the SB203580 (Figure 8(e)) and SP600125 (Figure 8(f)) treatment groups in liver I/R. These results indicated that P38/JNK MAPKs were located in the downstream of TLR4/HMGB1.

## 4. Discussion

Liver I/R was categorized into warm ischemia and cold ischemia; however, the two types share a common mechanism in the disease etiology, including primarily local damage and sequential systematic inflammatory cascade [28, 29] injury to the remote organs [30], especially to the lung. This noninfective inflammation in the lung activates various specific or nonspecific signaling pathways. This in turn aggravates the uncontrolled inflammatory responses, leading to systemic inflammatory response syndrome (SIRS), acute lung injury/acute respiratory distress syndrome (ALI/ARDS), and even to death [31, 32].

HMGB1 is considered as a late mediator compared with the release of other cytokines [33, 34], such as TNF and IL-1, in response to lethality during sepsis as well as after necrosis, but not apoptosis or death. However, Tsung et al. have exhibited that HMGB1 was upregulated early in the cultured hepatocytes during hypoxia and warm hepatic I/R in vivo [10]. TLR4 has been involved in I/R-induced inflammation in several organs in addition to the liver [35]. Our recent wok found that TLR4 combined with Wnt-induced secreted protein-1 (WISP1) participated in liver I/R injury [9]. However, TLR4 combined with HMGB1 participates in I/R injury, which is a traditional signaling pathway, and its involvement in the liver I/R-induced lung inflammation and injury is still unclear.

The purpose of our study was to test whether the first-generation HIV protease inhibitor, SQV, could inhibit TLR4/HMGB1 signaling to protect lung inflammation and injury induced by liver I/R. The major and novel findings of this investigation include the following: (a) SQV improved the lung structure injury, edema, and permeability induced by liver warm I/R; (b) SQV attenuated the expression of proinflammatory factors, such as IL-6, TNF-*α*, IL-1*β*, and iNOS, while upregulated the expression of anti-inflammatory factor, IL-10; (c) SQV decreased the circulating and lung BALF profactor levels, IL-6 and TNF-*α*, and enhanced the antifactor level of IL-10; (d) MIP-2 and I*κ*B*α* levels were regulated by SQV in the lung after liver I/R; in addition, the neutrophils displayed subtle changes, but macrophages as well as the apoptotic cells in lung tissue after SQV treatment were markedly decreased; (e) TLR4, HMGB1, and phosphorylated P38/JNK MAPKs were markedly suppressed by SQV in the lung tissues during liver I/R. Meanwhile, as HMGB1 can be released from both apoptotic cells and necrotic cells after liver IR, so, circulating HMGB1 levels were detected and significantly increased after liver I/R, but reversed by SQV treatment (Figure Supplement 1). These results provide evidence that SQV could fight against liver I/R injury.

To further study the mechanism of TLR4/HMGB1-P38/JNK MAPK pathway-mediated lung injury in hepatic I/R, we used TLR4^−/−^ mice as a vehicle to observe the changes of HMGB1 and phosphorylated P38/JNK in the lung tissues in liver I/R. Our results (Figure 7) showed that HMGB1 and phosphorylation of MAPK were significantly decreased. Furthermore, P38 and JNK inhibitors were administered in liver I/R wild-type mice (Figure 8) to demonstrate that MAPKs were present in the downstream inflammatory signaling pathway of HMGB1, which affected the degree of lung damage in liver I/R mice. Nevertheless, it is still unknown whether upregulation of HMGB1 is released from the liver through blood circulation or production by itself or both together. Though SQV could exert anti-inflammatory effect by the TLR4/HMGB1 MAPK signaling pathway, the in-depth relation between them needs further exploration. The receptor for advanced glycation end products (RAGEs) [20, 36] is also known as the receptor for HMGB1, and it plays a much important role in lung injury in liver I/R. In contrast, the ERK phosphorylation showed differences from P38 and JNK phosphorylation in liver I/R with/without SQV treatment. Hence, it is considered as an interesting point and needs further investigation.

## 5. Conclusion

Above all, our study demonstrated that SQV can attenuate lung injury induced by liver warm I/R via the HMGB1- and p38/JNK-mediated TLR-4-dependent signaling pathways.

## Figures and Tables

**Table 1:** Primers for quantitative polymerase chain reaction.

Gene	Primer	Sequence
TNF-*α*	Forward	5′-CCCTCACACTCAGATCATCTTCT-3′
	Reverse	5′-GCTACGACGTGGGCTACAG-3′
IL-1*β*	Forward	5′-GCAACTGTTCCTGAACTCAACT-3′
	Reverse	5′-ATCTTTTGGGGTCCGTCAACT-3′
IL-10	Forward	5′-GCTCTTACTGACTGGCATGAG-3′
	Reverse	5′-CGCAGCTCTAGGAGCATGTG-3′
iNOS	Forward	5′- GTTCTCAGCCCAACAATACAAGA-3′
	Reverse	5′- GTGGACGGGTCGATGTCAC-3′
HMGB1	Forward	5′-GGCGAGCATCCTGGCTTATC-3′
	Reverse	5′-GGCTGCTTGTCATCTGCTG-3′
TLR4	Forward	5′-AGGCACATGCTCTAGCACTAA-3′
	Reverse	5′-AGGCTCCCCAGTTTAACTCTG-3′
*β*-Actin	Forward	5′-GGCTGTATTCCCCTCCATCG-3′
	Reverse	5′-CCAGTTGGTAACAATGCCATGT-3′

## Supplementary Materials

Supplementary Materials Figure Supplement 1: circulating HMGB1 levels were tested by ELISA in all groups. SQV could effectively reduce the HMGB1 level after liver I/R. GraphPad values are presented as mean ± SEM. ^∗^ *P* < 0.05 versus the Sham group; ^#^ *P* < 0.05 compared to the liver warm I/R group.
